# A Video Self-Modeling Intervention Using Virtual Reality Plus Physical Practice for Freezing of Gait in Parkinson Disease: Feasibility and Acceptability Study

**DOI:** 10.2196/28315

**Published:** 2021-11-03

**Authors:** Lina Goh, Natalie E Allen, Naseem Ahmadpour, Kaylena A Ehgoetz Martens, Jooeun Song, Lindy Clemson, Simon J G Lewis, Hamish G MacDougall, Colleen G Canning

**Affiliations:** 1 Sydney School of Health Sciences Faculty of Medicine and Health The University of Sydney Camperdown Australia; 2 Sydney School of Architecture, Design and Planning The University of Sydney Camperdown Australia; 3 Department of Kinesiology Faculty of Health The University of Waterloo Waterloo, ON Canada; 4 Brain and Mind Centre The University of Sydney Camperdown Australia; 5 School of Psychology Faculty of Science The University of Sydney Camperdown Australia

**Keywords:** Parkinson disease, freezing of gait, action observation, video self-modelling, virtual reality

## Abstract

**Background:**

Despite optimal medical and surgical intervention, freezing of gait commonly occurs in people with Parkinson disease. Action observation via video self-modeling, combined with physical practice, has potential as a noninvasive intervention to reduce freezing of gait.

**Objective:**

The aim of this study is to determine the feasibility and acceptability of a home-based, personalized video self-modeling intervention delivered via a virtual reality head-mounted display (HMD) to reduce freezing of gait in people with Parkinson disease. The secondary aim is to investigate the potential effect of this intervention on freezing of gait, mobility, and anxiety.

**Methods:**

The study was a single-group pre-post mixed methods pilot trial for which 10 participants with Parkinson disease and freezing of gait were recruited. A physiotherapist assessed the participants in their homes to identify person-specific triggers of freezing and developed individualized movement strategies to overcome freezing of gait. 180° videos of the participants successfully performing their movement strategies were created. Participants watched their videos using a virtual reality HMD, followed by physical practice of their strategies in their own homes over a 6-week intervention period. The primary outcome measures included the feasibility and acceptability of the intervention. Secondary outcome measures included freezing of gait physical tests and questionnaires, including the Timed Up and Go Test, 10-meter walk test, Goal Attainment Scale, and Parkinson Anxiety Scale.

**Results:**

The recruitment rate was 24% (10/42), and the retention rate was 90% (9/10). Adherence to the intervention was high, with participants completing a mean of 84% (SD 49%) for the prescribed video viewing and a mean of 100% (SD 56%) for the prescribed physical practice. One participant used the virtual reality HMD for 1 week and completed the rest of the intervention using a flat-screen device because of a gradual worsening of his motion sickness. No other adverse events occurred during the intervention or assessment. Most of the participants found using the HMD to view their videos interesting and enjoyable and would choose to use this intervention to manage their freezing of gait in the future. Five themes were constructed from the interview data: reflections when seeing myself, my experience of using the virtual reality system, the role of the virtual reality system in supporting my learning, developing a deeper understanding of how to manage my freezing of gait, and the impact of the intervention on my daily activities. Overall, there were minimal changes to the freezing of gait, mobility, or anxiety measures from baseline to postintervention, although there was substantial variability between participants. The intervention showed potential in reducing anxiety in participants with high levels of anxiety.

**Conclusions:**

Video self-modeling using an immersive virtual reality HMD plus physical practice of personalized movement strategies is a feasible and acceptable method of addressing freezing of gait in people with Parkinson disease.

## Introduction

### Background

Parkinson disease is a progressive neurological condition that affects approximately 6 million people worldwide [1]. People with Parkinson disease can present with a variety of motor impairments such as tremor, slow movements, gait, and balance disorders, as well as nonmotor impairments such as reduced cognition, depression, anxiety, and sleep disorders [2]. Freezing of gait, defined as a brief, episodic, absence or marked reduction of forward progression of the feet despite the intention to walk, is a complex phenomenon that may also be present in people with Parkinson disease [3,4]. People with freezing of gait often describe their feet as being *glued to the ground*, which can lead to reduced mobility, falls, poor quality of life, and increased health care costs [4-8].

The pathophysiology underlying freezing of gait remains poorly understood, although it is suggested to result from dysfunction in neural networks across motor, affective, and cognitive domains [9-12]. People with freezing of gait are more likely to exhibit decreased gait automaticity and increased gait variability [13], as well as motor fluctuations and dyskinesia [14]. Furthermore, cognitive deficits and anxiety also appear to be more pronounced in people with freezing of gait than without [14-17]. Freezing of gait is also more frequent and severe in conditions of high levels of anxiety compared with low levels [18].

First-line treatments for freezing of gait consist predominantly of pharmacological interventions to maintain a good *on* state [4]. However, freezing of gait can persist despite these regimes. Nonpharmacological interventions are often used in conjunction with pharmacological interventions, with the most common being physiotherapy. Although several reviews have shown that these nonpharmacological interventions are effective, their results have been modest [19-23]. This might be because of the heterogeneity of the interventions, small study sample sizes, and limitations of relying on self-report to assess freezing of gait [21,24].

A recent systematic review performed subgroup analyses to determine which types of physiotherapy interventions may be the most useful in managing freezing of gait. The results showed that action observation training had a statistically significant effect on reducing freezing of gait [21]. In the 4 action observation studies, participants with freezing of gait watched videos of actors perform movement strategies designed to overcome freezing, followed by physical practice of these strategies under the supervision of a physiotherapist [25-28]. Observation of actions performed by others is understood to activate neural structures in the brain that execute the same actions, thus facilitating motor learning and performance [29]. This is then further reinforced by physical practice.

Although the results from these action observation studies were positive, the strength of this evidence was weak, and its clinical significance was unclear. This was because of the small number of moderate-quality studies included in the meta-analysis and the use of the New Freezing of Gait Questionnaire (NFOG-Q) [30] as the outcome, as this questionnaire was previously shown to be insufficiently responsive to detect small changes in freezing severity [24] and may not be a good indicator of the real symptom burden [31]. Furthermore, the implementation of action observation as an intervention for freezing of gait was limited in the following ways: (1) participants watched actors without Parkinson disease perform movement strategies, which might not be an accurate reflection of motor performance by people with Parkinson disease; (2) videos demonstrated generalized strategies that might not be relevant or appropriate for the individual; (3) videos showed the use of movement strategies to overcome freezing of gait in clinical settings, which might reduce ecological validity as patients typically present with freezing of gait at home [32]; and (4) participants watching videos on flat-screen devices could be vulnerable to distractions in their immediate environment, which might impact motor learning.

Video self-modeling, a form of observational learning that requires the observer to watch and learn from one’s own positive behavior, may be useful for people with Parkinson disease [33]. The activation of neural structures previously described is maximized when the observed actions are familiar to the observer and comprise movement that the observer is able to perform [29]. Therefore, we hypothesized that the use of video self-modeling would provide salient cues to improve motor learning and performance, as well as promote self-efficacy by strengthening beliefs in one’s ability to overcome freezing of gait [34]. People with freezing of gait may benefit further if they observe themselves using personalized strategies that address their specific motor, affective, and cognitive triggers of freezing. In addition, videos of situations at home that provoke freezing of gait (and strategies to successfully overcome freezing) may be of greater relevance. Furthermore, the use of a virtual reality head-mounted display (HMD) removes distractors in the environment, directing full attention to viewing videos.

### Objective

Therefore, the aim of this study is to investigate the feasibility and acceptability of a home-based, personalized video self-modeling intervention delivered via a virtual reality HMD to reduce freezing of gait in people with Parkinson disease. The secondary aim is to investigate the potential effect of this intervention on freezing of gait, mobility, and anxiety.

## Methods

### Design

A single-group pre-post mixed methods pilot trial was conducted from April 2019 to April 2020. Ethics approval was obtained from the University of Sydney Human Research Ethics Committee (project number 2018/893), and written informed consent was obtained from all participants. The trial was registered with the Australian New Zealand Clinical Trials Registry (ANZCTR12619000139178).

### Participants

A total of 10 participants were recruited from existing databases of people with Parkinson disease at the University of Sydney and from Parkinson’s New South Wales support groups. The following inclusion criteria were used: (1) diagnosis of idiopathic Parkinson disease, (2) presence of freezing of gait (defined as having a score of ≥1 on question 2 and score of ≥2 on question 4 of the NFOG-Q), (3) stable dopaminergic medication regime for at least 4 weeks before commencing the study, (4) ability to walk independently with or without a walking aid, and (5) living in the greater Sydney metropolitan area. Participants were excluded if they had (1) any medical conditions that would interfere with the study safety and conduct, such as unstable cardiovascular disease and neurological conditions other than Parkinson disease; (2) cognitive impairment defined as having a score of <24 on the Mini Mental State Examination [35]; (3) newly commenced deep brain stimulation or changes in stimulation parameters within 6 months before participating in the study; and (4) significant head tremor or motion sickness limiting the ability to use a virtual reality HMD.

### Intervention

**Figure 1:**
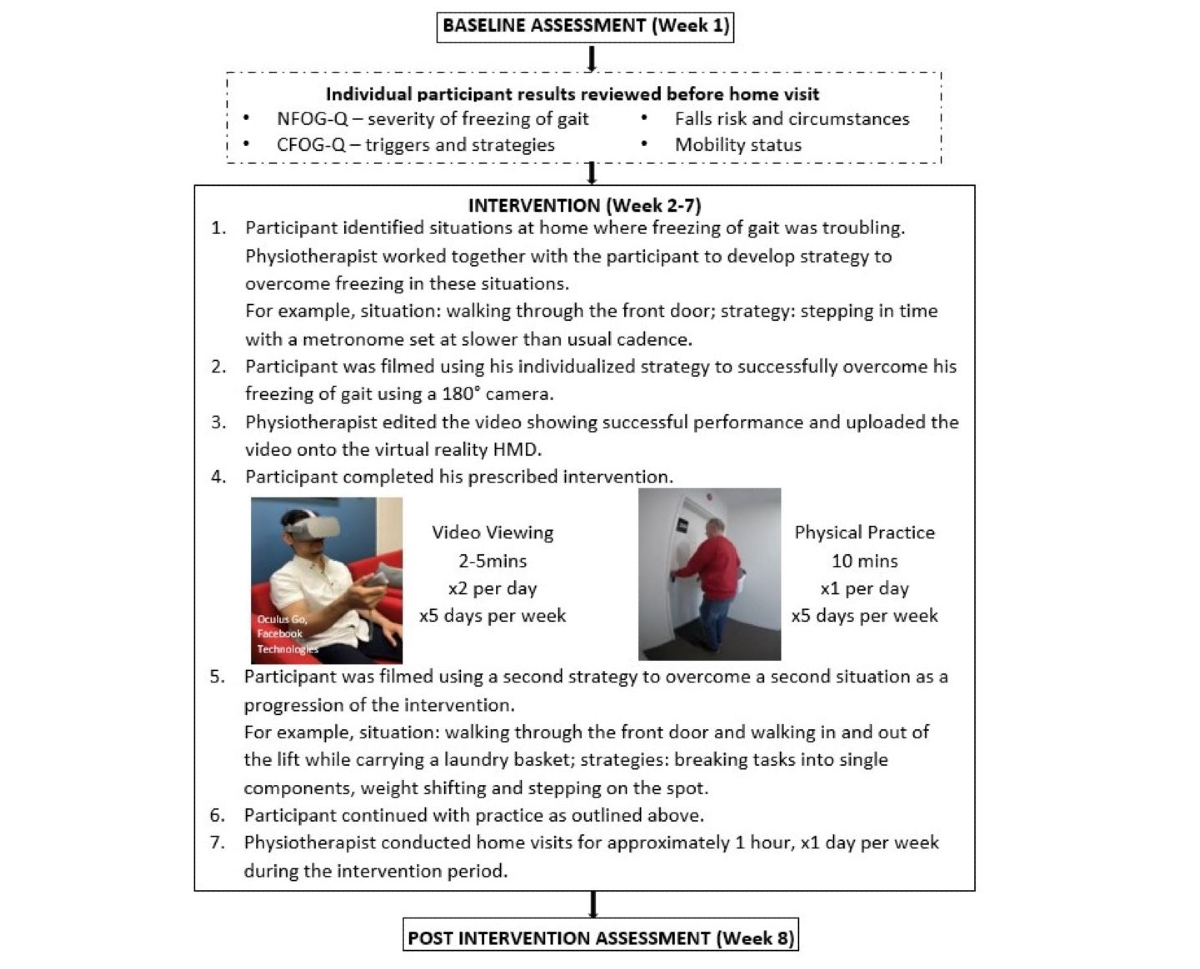
Example of the intervention. CFOG-Q: Characterizing Freezing of Gait Questionnaire; HMD: head-mounted display; NFOG-Q: New Freezing of Gait Questionnaire.

The intervention protocol (including printed information and instructions to the participants) is described in detail in Multimedia Appendix 1 [23,25,36-38] and illustrated in Figure 1. In brief, a physiotherapist (LG) delivered 6-8 home visits over 6 weeks, with each visit lasting approximately 60 minutes. The interventions were delivered, and practice was completed when participants were in their *on* phase, that is, when their medications were working optimally.

Before the first home visit, the physiotherapist reviewed the participants’ medical history, including disease and freezing of gait severity (NFOG-Q), mobility status, and the circumstances of any falls. The physiotherapist also evaluated the baseline results of participants’ Characterizing Freezing of Gait Questionnaire (CFOG-Q) to determine their personal motor, cognitive, emotional, and environmental triggers of freezing of gait, as well as any strategies previously used to overcome freezing [36].

During the first home visit, participants identified a situation at home, where freezing of gait was troubling. Examples of situations included turning around in tight spaces and walking through doorways while performing an additional motor task. The physiotherapist assessed the participants’ performance in this situation and worked with participants to develop a suitable personalized strategy to help them overcome their freezing. Examples of strategies included stepping in time to the beats of an external rhythmic auditory cuing device, such as a metronome typically set at lower than usual cadence; self-initiated movement strategies, such as counting, shifting weight from side to side, and/or simplifying complex tasks; and progressing by increasing the complexity of tasks where appropriate.

Once a situation was identified and a strategy developed and practiced by the participants, a 3D camera (Mirage, Lenovo) was used to produce 180° videos showing the participants using their personalized strategy to successfully overcome freezing of gait in the situation identified. The videos were edited to show the participants’ successful performance 3 times and lasted 2-5 minutes. The videos were created to be compatible with a virtual reality HMD (Oculus Go, Facebook Technologies). Participants were instructed on the use of the HMD and asked to watch their personal video using the HMD while seated twice a day, 5 days per week for 6 weeks. After one of the 2 daily video viewings, participants performed the physical practice of their strategy, as shown in their video for 10 minutes. Participants were also provided with a printed guide on how to use and navigate the virtual reality system.

The physiotherapist monitored participants’ progress during home visits. Once participants were able to consistently use their strategy to overcome freezing of gait in the first situation, a second situation was identified, and a second strategy was developed. A second video was created for each participant. This second video involved the participant performing the same task in a different environment, the same task with increased complexity, or a different task altogether. Strategies used to overcome freezing of gait were adjusted as appropriate depending on the situation, the person-specific freezing of gait triggers, and preferences. Participants were then asked to watch their second video and perform its associated physical practice 4 times a week, and the first video and its associated physical practice once a week. If participants continued to make further progress, a third situation was introduced, and this process continued for the duration of the intervention period. The introduction of additional videos was determined by the physiotherapist and was based on clinical judgment and participants’ self-assessment of their progress.

### Outcome Measures

The primary outcome measures assessed the feasibility and acceptability of the intervention. Measures of feasibility included recruitment rate, retention rate, and adherence to the intervention (by recording the number of daily video viewings and physical practice) using self-report logbooks and adverse events associated with the intervention. Measures of acceptability included a modified Players Experience of Need Satisfaction (PENS) Questionnaire [39] and a semistructured interview at postintervention. In the modified PENS Questionnaire (Multimedia Appendix 2 [39]), participants were asked to reflect on their experience of using the virtual reality system, which comprised the HMD, the handheld control, and the experience of navigating and viewing the videos. Participants rated their interest or enjoyment, sense of presence or immersion, competence, and intuitiveness of the controls and rated 2 additional items that were added to the PENS. These were the presence of motion sickness and whether they would use the virtual reality system for the management of their freezing of gait in the future if it was available. The questionnaire included 18 questions across the categories listed above, with each question scored on a 7-point Likert scale, where 1=strongly disagree and 7=strongly agree (higher score is better). In the interviews, participants were asked to describe their experiences of the intervention, including both video viewing and physical practice (Multimedia Appendix 3).

Secondary outcome measures were collected to assess any potential effects of the intervention on freezing of gait, mobility, and anxiety. These measures were collected at baseline and postintervention. Participants also completed the Goal Attainment Scale, with goals in relation to managing freezing of gait at home set at baseline and goal attainment evaluated at postintervention [40].

To obtain freezing of gait measures, participants were videotaped by performing 2 freezing of gait provoking tests: the Ziegler test [41] and the turn-in-place test [42]. In the Ziegler test, participants began in a seated position 3.4 m from a closed door. Participants were asked to stand up, walk forward 1 m to a square outlined with tape on the ground (40×40 cm), perform two 360° turns (clockwise and counter-clockwise) within the square, and walk forward a further 2 m to open the door and walk through the doorway, before returning to sit in the chair. Participants were asked to perform the test as fluently as possible under 3 conditions in the following order: (1) no additional task, (2) with an additional motor task (ie, carrying a tray with a cup of water), and (3) with additional motor and cognitive tasks (ie, carrying a tray with a cup of water and counting backwards by 7 from 100). In the turn-in-place test, participants were asked to turn 360° on the spot, alternating right and left at a self-selected pace for 1 minute.

Two assessors (KAEM and JS) determined the percentage of time frozen for each freezing of gait provoking test, plus the time taken to complete the Ziegler test, via offline video analyses [43,44]. They were blinded to the baseline and postintervention testing conditions. A detailed protocol is described in Multimedia Appendix 4 [4,43]. Interrater reliability between the assessors was excellent. The intraclass correlation coefficients (two-way mixed effects, absolute agreement) were as follows: Ziegler test percent time frozen=0.962 (from analyses of 29 videos), Ziegler test duration=1.000 (from analyses of 29 videos), and turn-in-place test percent time frozen=0.984 (from analyses of 20 videos). The videos used to determine intraclass correlation coefficients were from baseline *on* and *off* performances of 5 participants where postintervention measures were not available.

Participants also completed the NFOG-Q, which reports the severity and impact of freezing of gait (range 0-28), and the CFOG-Q, where section 2 reports the frequency of freezing of gait triggers (range 0-48). Lower scores indicate less severe freezing of gait in both measures.

The mobility measures included comfortable walking speed (measured over 10 m) [45] and the Timed Up and Go Test [46] in single- and dual-task conditions (ie, counting backward from 100 by 3), with lower scores indicating better mobility. Anxiety was measured using the Parkinson Anxiety Scale (PAS; range 0-48), with lower scores indicating lower levels of anxiety [47].

Participants were assessed at baseline within a week before the start of the intervention and postintervention within a week of completing the intervention. The following demographic information was also collected at baseline: age, gender, severity of Parkinson disease using the Movement Disorder Society—Unified Parkinson’s Disease Rating Scale Section III [48] and the Hoehn and Yahr stage [49], cognitive function using the Trail Making Tests A and B [50], and current medication regimen. Assessments were conducted at a university laboratory when participants were in their *on* phase. The freezing of gait provoking tests (both Ziegler and turn-in-place tests) were also repeated in participants’ homes when they were in their *off* phase after 12 hours withdrawal of their levodopa medication overnight.

### Adverse Events

The presence of motion sickness experienced while using the virtual reality HMD and any falls, injuries, and fatigue during the intervention and assessments were monitored. Participants were asked to record any adverse events in a logbook and report the events to the researchers. At each home visit, the researcher questioned the participants about the occurrence of any adverse events.

### Statistical Analyses

Descriptive statistical analyses of feasibility and secondary outcome measures were conducted using SPSS Statistics for Windows, version 26.0 (IBM Corporation). Interview data were audio-recorded and transcribed verbatim by independent transcribers who were external to the study. NVivo 12 software (version 2, QSR International) was used to code the interview data, and inductive thematic analysis was used to interpret the results [51]. One researcher coded all of the interviews (LG) and 2 other researchers coded parts of the interviews (CGC and NA) such that all data were coded independently by at least 2 researchers. The codes were then compared and grouped to form the main themes through an iterative process. Any differences were discussed in depth until a consensus was reached among the 3 researchers.

## Results

### Overview

Individual and aggregate participant background information is presented in Table 1. Overall, participants (9 males and 1 female) had a mean age of 70.6 years (SD 7.7 years), were diagnosed with Parkinson disease for an average of 13.3 years (SD 5.2 years), and had moderate to severe disease severity with a mean Movement Disorder Society—Unified Parkinson’s Disease Rating Scale Section III score of 37.3 (SD 13.3) [52]. All participants had moderate to severe freezing of gait (NFOG-Q range 10-24/28), and 5 participants had significant anxiety (PAS>14/48). A total of 6 participants were considered recurrent fallers (defined as having more than 2 falls in the past 12 months), with 2 participants experiencing particularly high rates of falls. A total of 2 participants had not fallen in the past 12 months (Table 1).

**Table 1:** Baseline characteristics of participants.

	Age (years)	Parkinson disease duration (years)	Movement Disorder Society—Unified Parkinson’s Disease Rating Scale Section III (0-132)^a^	Hoehn and Yahr scale (1-5)^a^	Mini Mental State Examination (0-30)^b^	Trail Making Test part A (seconds)^a^	Trail Making Test part B (seconds)^a^	New Freezing of Gait Questionnaire (0-28)^a^	Parkinson Anxiety Scale (0-48)^a^	Timed Up and Go Test (seconds)^a^	Number of falls in the past year	Levodopa equivalent daily dose (mg)
P1	81	12	53	3	26	79.0	351.0	22	14	27.0	4	1120
P2	62	14	18	2	30	14.9	26.9	24	9	10.4	1	2083^c^
P3^d^	61	18	40	3	30	18.9	49.9	22	10	12.9	6	724
P4	78	11	29	2	30	49.0	169.0	15	10	12.3	6	1300
P5	76	5	45	2	29	13.0	130.2	11	11	12.9	1	600
P6	75	8	27	2	28	59.0	106.0	17	18	13.9	0	900
P7	66	16	35	3	25	41.4	284.5	23	29	15.3	Approximately 550^e^	600^f^
P8	60	10	47	2	29	62.0	87.0	24	40	14.5	6	1048
P9^g^	72	23	22	3	28	28.6	95.0	21	3	9.4	Approximately 230^h^	1694
P10^g^	75	16	57	2	28	49.4	149.9	21	20	14.0	0	400
Value, mean (SD)	70.6 (7.7)	13.3 (5.2)	37.3 (13.3)	2.4 (0.5)	28.3 (1.7)	41.5 (22.2)	144.9 (101.7)	20.0 (4.3)	16.4 (11.0)	14.3 (4.8)	N/A^i^	1046.9 (527.6)

^a^Lower scores are better.

^b^Higher scores are better.

^c^This participant is on Duodopa therapy.

^d^This participant withdrew because of medical reasons unrelated to the trial.

^e^This participant reported 1 to 2 falls per day.

^f^This participant received deep brain stimulation.

^g^This participant did not receive the complete intervention because of COVID-19 lockdown.

^h^This participant reported 4 to 5 falls per week.

^i^N/A: not applicable.

### Primary Outcome Measures

#### Feasibility

**Figure 2:**
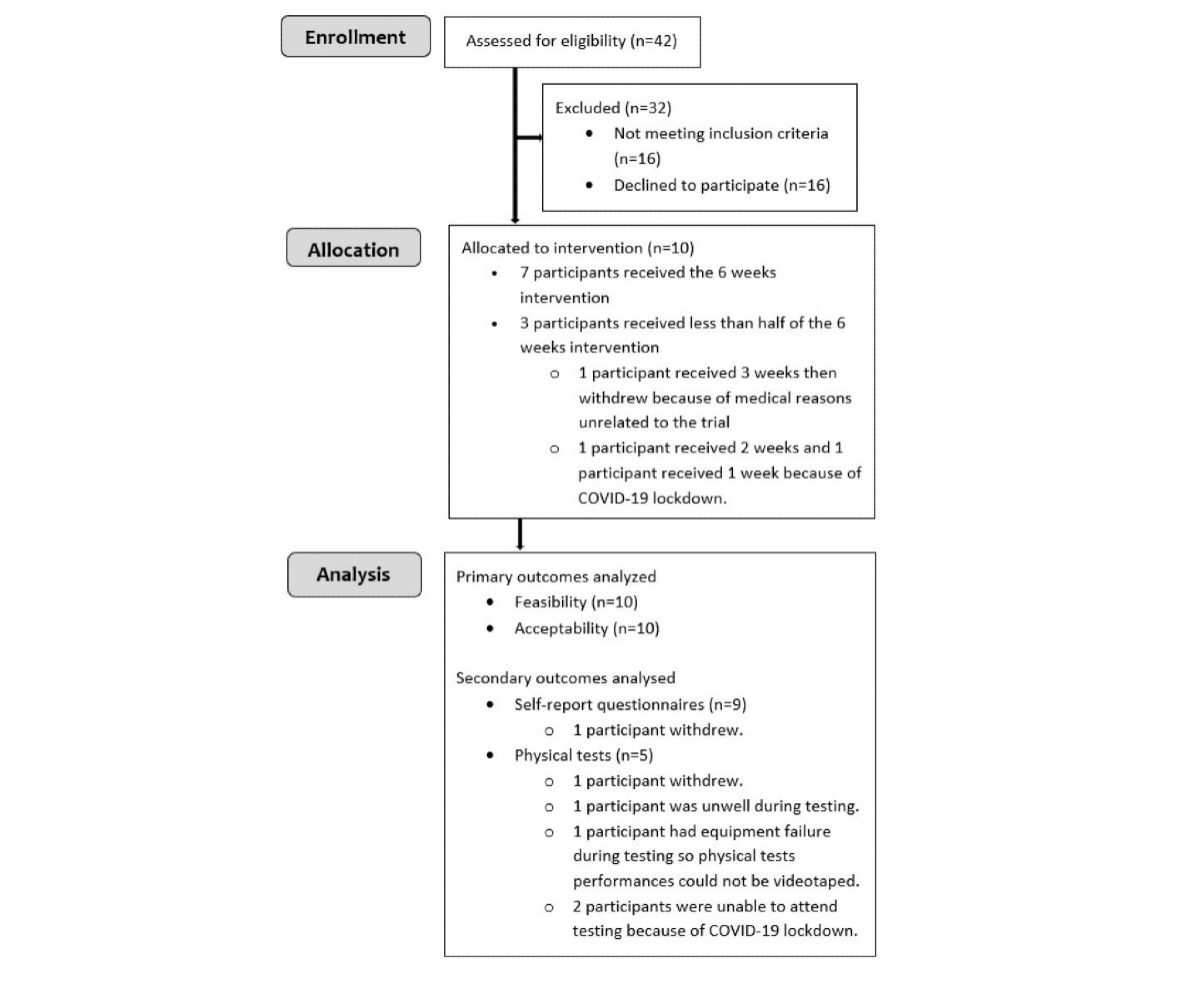
Flowchart of participants through the study.

The flow of the participants in this study is shown in Figure 2. A total of 42 potential participants were identified and assessed for eligibility. Of these 42 participants, 10 participants consented to participate, resulting in a 24% (10/42) recruitment rate. A total of 1 participant withdrew after receiving 3 weeks of intervention because of medical reasons unrelated to the study, resulting in a 90% (9/10) retention rate.

All primary and secondary outcome measures were obtained from 10 participants at baseline. At postintervention, all primary outcome measures were obtained, but only some secondary outcome measures were available. A total of 9 participants completed the subjective questionnaires, and 5 completed all the in-person physical tests. In addition to the participant who withdrew from the study, 1 participant became unwell during testing; 1 participant had equipment failure during testing, so physical test performances could not be videotaped; and 2 participants were unable to attend in-person testing because of a city-wide lockdown from the COVID-19 pandemic.

Adherence to the intervention was good, with participants completing a mean of 84% (SD 49%; range 8%-153%) of the prescribed video viewing and a mean of 100% (SD 56%; range 17%-187%) of the prescribed physical practice (Table 2). A total of 2 participants received less than 2 weeks of supervised home visits as a result of the COVID-19 lockdown. A total of 1 participant was unable to tolerate the use of the virtual reality HMD because of dyskinesia of the head and neck, resulting in dizziness when viewing his videos. He used the HMD for 1 week and completed the rest of the intervention using a flat-screen device. No other adverse events occurred during the intervention or assessment.

**Table 2:** Adherence to the intervention.

	Video viewings (% completed)	Physical practice (% completed)
P1	105	107
P2	143	143
P3^a^	18	23
P4	53	50
P5	105	113
P6	153	187
P7	73	97
P8^b^	117	157
P9^c^	65	107
P10^c^	8	17
Value, mean (SD)	84 (49)	100 (56)

^a^This participant withdrew because of medical reasons unrelated to the trial.

^b^This participant completed the majority of the intervention using a flat-screen device.

^c^This participant did not receive the complete intervention because of COVID-19 lockdown.

#### Acceptability

Results from the modified PENS Questionnaire (Tables 3 and 4) suggested that the majority of participants found the use of the virtual reality system to view their videos interesting and enjoyable. Participants experienced minimal motion sickness and would choose to use the virtual reality system to manage their freezing of gait in the future. Results for competence, presence or immersion, and intuitive controls were overall positive but variable between participants.

**Table 3:** Results from the modified Players Experience of Need Satisfaction Questionnaire.

Modified Players Experience of Need Satisfaction Questionnaire question		P1	P2	P3	P4	P5	P6	P7	P8	P9	P10
**Interest or enjoyment**											
	I enjoyed doing this activity very much.	7	6	4	5	4	7	7	7	7	5
	This activity was fun to do.	7	5	4	6	3	7	7	6	7	5
	This was not a boring activity^a^.	2	3	6	7	3	6	7	7	7	7
	This activity did hold my attention^a^.	6	2	6	7	5	7	7	6	7	6
	I would describe this activity as very interesting.	6	5	5	5	4	7	7	6	7	6
	While I was doing this activity, I was thinking about how much I enjoyed it.	4	5	4	5	5	4	7	5	4	2
**Competence**											
	I felt competent at using the virtual reality system to watch my videos.	6	7	5	4	5	6	6	2	7	2
	I felt very capable and effective when using the virtual reality system.	6	7	5	3	6	6	6	2	7	2
**Presence or immersion**											
	When watching my videos, I felt transported to another time and place.	1	5	2	6	6	4	7	2	1	5
	When watching my videos, I felt as if I was actually there.	5	5	5	4	6	6	7	5	7	5
	Watching my videos was engaging.	6	5	4	5	5	6	7	5	4	5
	I experienced feelings as deeply in my videos as I have in real life.	4	4	3	5	5	3	7	5	4	3
	I experienced genuine pride when I watched my videos.	4	4	2	4	3	6	2	6	4	1
	I did not experience distress when I watched my videos^a^.	7	7	6	7	6	7	1	4	7	7
**Intuitive controls**											
	Learning to use the virtual reality system was easy.	3	7	5	4	5	3	6	3	7	2
	The virtual reality system controls were intuitive.	3	7	4	4	5	4	6	3	4	2
**Future participation**											
	If given the opportunity, I would use virtual reality systems for the management of my freezing of gait in the future.	5	7	2	5	5	4	7	6	7	7
**Motion sickness**											
	I did not experience motion sickness while using the virtual reality system^a^.	7	7	6	7	6	7	7	5	7	7

^a^This question was rephrased so that its effect direction is similar to that of the other questions to allow for the calculation of mean and SD (Multimedia Appendix 2).

**Table 4:** Overall results from each category from the modified Players Experience of Need Satisfaction Questionnaire^a^.

Category	Value, mean (SD)
Interest or enjoyment	5.6 (1.5)
Competence	5.0 (1.8)
Presence or immersion	4.7 (1.7)
Intuitive controls	4.4 (1.6)
Future participation	5.5 (1.6)
Motion sickness	6.6 (0.7)

^a^Overall mean: 5.3 (SD 1.5).

All the participants completed semistructured interviews after the intervention. Each interview lasted approximately 45-60 minutes.

Five themes emerged from the interviews. An overview of these results is presented below, whereas codes and further supporting quotes for each theme are presented in Multimedia Appendix 5.

1. Reflections when seeing myself: while most participants were embarrassed or shocked by their appearances in the videos, these feelings motivated them to change. These participants used the videos to identify areas of improvement to correct their posture and gait. A total of 1 participant, who wanted to hide his disease from others, was disappointed, as he felt that he was not achieving this as well as he thought. A total of 2 participants were unconcerned with their appearances and solely focused on using the videos to learn the movement strategies to help them overcome their freezing of gait:

One of the things that I changed, looking at the video, my posture and my walking, struck me as pretty poor. I have to make conscious decision, effort, to improve.

P5

2. My experience of using the virtual reality system: a total of 5 participants needed assistance from their carers to don or doff the HMD and navigate the platform to view their videos. Participants felt frustrated if they could not easily access their videos or when they made mistakes when using the controller to make selections. Although their abilities to use the system improved with practice, frustration when using the HMD decreased participants’ motivation to engage with the intervention:

It strikes me that the virtual reality system is not foolproof; if you make a mistake in one menu or something, you have to find your way back. And any frustration like that, you don't want to carry on.

P10

On the other hand, some participants found using the system easy and straightforward. Pre-existing technological literacy in participants might account for individual experiences when using the virtual reality system. Participants who reported feeling comfortable using technological devices found it easy to use the virtual reality system. In contrast, participants who were unfamiliar and not confident with technology reported difficulties.

Although participants were provided with personalized instruction and a printed user guide, most participants requested further resources and support to use virtual reality, such as video tutorials.

3. The role of the virtual reality system in supporting my learning: most participants found the use of the virtual reality system to be beneficial. The virtual reality system was seen as a novel and engaging tool to learn movement strategies. Using the HMD minimized distractions in the environment and allowed participants to concentrate on viewing the videos. However, a total of 2 participants did not perceive the virtual reality system as superior to flat-screen devices such as tablets, stating cost and usability as barriers to using the system:

There're less outside influences to bother you. Because if I'm looking at a computer screen...I mean there're other things on the desk, on the table, in the background. Whereas this headset excludes all that out of the equation.

P9

I don’t think it’s any better than say someone holding a phone or a video and watching, so if there is a cost to it which obviously there is quite a considerable amount of money involved.

P3

4. Developing a deeper understanding on how to manage my freezing of gait: all participants found that viewing their videos was helpful. Most participants used their videos as a guide to learn how to overcome their freezing of gait, whereas 1 participant used his video to work out how to hide his Parkinson disease. Several participants valued viewing themselves in the videos as it best reflected their individual disease presentation and personalized strategies to overcome their freezing. Participants also saw videos as exemplars for physical practice:

You got that the video of what you’re supposed to be doing as you are doing it. You tell me what to do, and I didn’t have the video, I don’t know—I might think I’m doing it the right way. And you come back in a week’s time and say that’s not what I meant at all. So I’m wrong—if I have the video it shows me what I’m meant to do.

P2

The person you can most identify with. And that's yourself. The most appropriate person is the person who's got Parkinson's themselves and is functioning. And you can see what the program is doing for you, and with you.

P9

Most participants found the video viewing and physical practice repetitive but worthwhile. Overall, repetitive practice was acceptable as a way of reinforcing good performance, and participants reported feeling better equipped to overcome their freezing of gait when it was triggered. However, repetitive physical practice appeared to be more accepted by participants than repetitive video viewing. Several participants suggested reducing the amount of video viewing, especially once they had mastered the movement strategies. None of the participants reported issues related to the amount of physical practice:

I think it's probably the repetitiveness that is helping you without you being aware. The fact that you have done it so many times makes it easier.

P6

I think it prepares more the attitude to when (freezing of gait) happens. For instance, you don’t cut it completely or diminish. But when it happens, you get out of the situation much quicker.

P5

5. Impact of the intervention on my daily activities: several participants reported improvements in their ability to manage their freezing of gait in other tasks and contexts. In total, 1 participant reported an improved ability to manage his freezing of gait outside of his home. He was able to board and alight from a ferry after having previously avoided public transportation. A total of 2 participants reported an improved ability to manage their freezing of gait when they were off their Parkinson disease medication. Participants reported being more confident and less anxious when they performed tasks that typically triggered their freezing of gait:

I've got more confidence. I don't panic as bad as I used to. I'm not afraid to walk around the house or outside. This is a really great help.

P8

In the freezing, yes. I experienced also, improvement. I got, the times that I got more intense, or the freezing when I wake up at night and I’m not with the medicine obviously. At that moment, almost every time is freezing but I start I stomp on the floor a little bit to start walking and so I applied the technique.

P5

You know, this is a tool. By using it and doing things, (P7) can improve the way he does things around the house. The amount of falls he's had, I think have been reduced. His confidence has been a lot better.

P7 carer

### Secondary Outcome Measures

#### Overview

A total of 6 participants achieved their freezing of gait goal or reported performing somewhat better than their goal after receiving the intervention. A total of 3 participants reported no change. Of these, 2 participants did not receive complete intervention because of the COVID-19 lockdown (Table 5).

**Table 5:** Goal Attainment Scale.

ID	Goal	Performed worse than baseline (-2)	Performed at baseline level of performance (-1)	Achieved goal as expected (0)	Performed somewhat better than expected (1)	Performed much better than expected (2)
P1	Turning around to sit on a chair, toilet, or car			✓		
P2	Walking out of the unit through the front door				✓	
P3^a^	Turning and stepping backwards at night					
P4	Turning around to sit down on the toilet (tight space)				✓	
P5	Taking the first step after standing up			✓		
P6	Walking from the bathroom to the kitchen when I am OFF my medication			✓		
P7	Walking in and out of the bathroom and laundry (tight space)				✓	
P8	Turning when opening or closing the fridge		✓			
P9^b^	Experiencing freezing of gait in the study		✓			
P10^b^	Experiencing freezing of gait in the toilet		✓			

^a^This participant withdrew because of medical reasons unrelated to the trial.

^b^This participant did not receive the complete intervention because of COVID-19 lockdown.

Overall, there were minimal changes to the freezing of gait, mobility, or anxiety measures from baseline to postintervention. However, there was substantial variability among participants (Multimedia Appendix 6). As this pilot study was not sufficiently powered to detect pre-post group differences, the results of 2 participants who were similar at baseline but demonstrated different levels of engagement with the intervention are highlighted below in more detail.

#### Participant 6

P6 was a 75-year-old female with mild to moderate disease. She had good cognition and mobility but significant freezing of gait, which was predominantly during her *off* phase. P6 also reported high levels of anxiety. She was highly engaged and responded well to the intervention, with multiple positive outcomes.

P6 viewed and practiced 2 different tasks. In her first video, she walked from her bathroom to her kitchen. This involved making a number of turns (including a 180° turn), navigating 2 doorways, and walking along a narrow passageway. The strategy she implemented was stepping in time to a metronome at 80 beats per minute (70% of her usual cadence measured in her baseline 10-meter walk test). This strategy was selected because she reported enjoying dancing and music and had previously responded well to rhythmic music. In her second video, she practiced a more complex task of making a cup of tea in her kitchen. This involved making a number of 180° turns while performing dual tasks, that is, holding a cup. The strategy that she implemented was counting her steps (*one, two, one, two, ...*), especially during turning. The metronome was not used in this task because of multiple noncontinuous segments. Dual tasks were also simplified into single tasks, where P6 would move the cup of tea along the bench top and take sideway steps separately so that she did not have to walk and hold onto a hot beverage concurrently (Multimedia Appendix 7).

Overall, P6 had a deep understanding of the rationale for the intervention. During her interview, she reported:

Because I visualized it, I watched it first, and it was in my head, it made it easier when I got up to move along doing the same actions. In that way it helped when I actually had to do it. I just sort of didn’t have to think. Because it was like in my head. And so I just moved, you know, the way I actually visualized it.

P6 also recognized the importance of repetitive practice. She demonstrated high levels of adherence to the intervention and completed more practice than what was prescribed (video viewing=153%; physical practice=187%). Although she did not consider the virtual reality system easy or intuitive to use (PENS intuitive control=3.5/7), she felt competent using the system (PENS competence=6.0/7) and reported using the system to view her videos as interesting and enjoyable (PENS interest or enjoyment=6.3/7).

Her goal was to reduce the severity of her freezing of gait when walking from her bedroom to her kitchen before her first dose of Parkinson disease medication every morning, and she reported achieving this goal after the intervention. At postintervention, she had a reduction in percent time frozen (from 94% to 84%) and time taken to complete the Ziegler test (from 489 s to 393 s) during *off* testing. She also had a significant reduction in her anxiety score (PAS from 18/48 to 3/48), and her freezing of gait was triggered less often (CFOG-Q from 31/48 to 17/48).

#### Participant 4

P4 was a 78-year-old male with mild to moderate disease. He had good mobility but reported significant freezing of gait, which was also predominantly during his *off* phase. Compared with P6, he had poorer executive function (based on the Trail Making Test part B) [53] and had lower levels of anxiety. He appeared less engaged and did not respond well to the intervention.

P4 viewed and practiced 3 similar but progressively more difficult tasks. In his first video, he practiced getting on and off the toilet, which involved walking and turning in a narrow space. In his second video, he practiced getting on and off a low, soft, and deep couch. In his third video, he practiced standing up and walking away from a chair at his desk. In all 3 videos, he used movement strategies of counting his steps (*one, two, one, two, ...*) and marching on the spot when turning. P4 had not tried any movement strategies before this study, and these strategies were selected as he found them easy to implement.

Although he recognized that the movement strategies were helpful and valued seeing himself in his videos so that he could improve his posture and walking, P4 had a low level of engagement with the intervention overall. He did not feel competent using the virtual reality system (PENS competence=3.5/7) and reported experiencing difficulties using the virtual reality system in his interview:

Because I had these problems, I sort of I don’t know how to fix it so I’ll wait till (the physiotherapist) comes or ring (the physiotherapist).

He also demonstrated low levels of adherence to the intervention (video viewing=53%; physical practice=50%). Interestingly, he stated several times he wished there were more applications on the virtual reality system he could explore:

I would have liked to, I think you can play games on it, can’t you?

His goal was to reduce the severity of his freezing of gait when turning to sit down on the toilet, and he reported achieving this goal somewhat better than expected after the intervention. His perception of his performance contrasted with his postintervention outcome measures, as there were small increases in percent time frozen (from 22% to 27%) and time taken to complete the Ziegler test (from 37 s to 51 s) during *off* testing. His other freezing of gait measures and his anxiety also increased (NFOG-Q from 15/28 to 27/28, CFOG-Q from 12/48 to 21/48, and PAS from 10/48 to 19/48).

## Discussion

### Principal Findings

To the best of our knowledge, this pilot study is the first of its kind to examine the feasibility and acceptability of video self-modeling (delivered via a virtual reality HMD) plus physical practice to help people with Parkinson disease manage their freezing of gait. The results of this study showed that the intervention is a feasible and acceptable option for addressing freezing of gait in people with Parkinson disease.

This intervention was safe to deliver with no participants experiencing falls during the intervention or assessments, despite freezing of gait, meaning that participants were at a high risk of falls [37]. The physiotherapist who delivered the intervention supervised the physical practice during the home visits until the participant was deemed safe to practice either independently or with a trained carer. Future larger randomized studies of video self-modeling plus physical practice to address freezing of gait should consider investigating falls as an outcome given the potential for this intervention to ameliorate fall risk.

As virtual reality motion sickness (due to sensory conflicts between visual, vestibular, and proprioceptive inputs) was a possible adverse event, susceptibility to motion sickness was an exclusion criterion. Although all participants were screened for motion sickness, 1 participant gradually developed intolerance of the HMD after 1 week. This was because of an exacerbation of his head and neck dyskinesia when wearing the HMD, which contributed to motion sickness. The camera and virtual reality system used in this study were commercially available devices selected for their accessibility to clinicians and relatively low cost. Although virtual reality motion sickness may be minimized by improving the technical aspects of the viewing experience, such as 6 degrees of freedom tracking, using dynamic depth of field, and providing multimodal sensory information [54], these improvements would require further development and involve higher costs. Future research to determine the incidence and intensity of virtual reality motion sickness, as well as the impact of varying technical aspects and costs of virtual reality systems, is needed in this population. Nevertheless, the use of a flat-screen device was found to be acceptable to the participant who required it.

The retention rate and adherence to intervention were high in this study. Participants completed most of the prescribed video self-modeling and physical practice sessions, with adherence rates comparable with those of other home-based exercise interventions [55]. Interestingly, the adherence rate of video viewing was less than that of physical practice. It was likely that repetitive viewings of the videos were more tedious compared with physical practice and that participants placed more value on physical practice over video viewing, even though both aspects of this intervention were designed to be complementary. This interpretation is supported by interview data, where several participants suggested reducing the amount of video viewing but did not suggest reducing the amount of physical practice. The amount and distribution of video viewing and physical practice should be further investigated in future studies to determine the best combination to improve performance while remaining engaging.

When comparing the recruitment rate of this study with others specifically targeting freezing of gait [28,56-59], our recruitment rate was lower. This was likely because of our recruitment from a general database of people with Parkinson disease, where a large proportion of individuals did not meet the inclusion criteria for this pilot study. It is also possible that potential participants with freezing of gait were deterred by the time needed to commit to the study and unfamiliarity with the technology.

This intervention was widely accepted. Most of the participants considered the intervention appropriate for their needs and would choose to do this again in the future. Although video self-modeling may be a useful tool for learning movement strategies, its use in people with Parkinson disease should be carefully considered. When people with Parkinson disease view themselves, they can bring up powerful emotions that are both positive and negative. Video self-modeling may improve self-efficacy by providing an opportunity for individuals to see their potential to overcome their freezing of gait. On the other hand, other forms of observational learning may need to be considered for people with Parkinson disease who are uncomfortable viewing themselves. Future studies are required to determine whether video self-modeling plus physical practice is superior to more generic observational learning plus physical practice for addressing freezing of gait.

Difficulties operating and navigating the virtual reality system to view videos may hinder the learning of the movement strategies and reduce adherence to the intervention. People with Parkinson disease may have tremors and dyskinesia, which makes it difficult to use a small controller, especially when vision is eliminated. They may also have difficulties donning and doffing an HMD that requires multiple adjustments to fit securely. In addition, people with significant cognitive impairments are likely to have issues using and engaging with a complex virtual reality system. Simple and intuitive platforms that provide people with Parkinson disease with large icons for selection and sufficient time to respond facilitates navigation of the applications.

Overall, the interpretation of secondary outcomes was limited by the small sample size and the variability of the results. Although there were minimal changes in the severity of freezing of gait, this intervention may be effective in reducing anxiety in participants with high levels of anxiety. Of the 5 participants with significant levels of anxiety at baseline (PAS>14/48), 4 participants (P6, P7, P8, and P10) had significant reductions in their anxiety scores. One additional participant (P2), who did not meet the cut-off for significant anxiety, also demonstrated a significant reduction in his anxiety score (PAS from 9/48 to 0/48). This finding is supported by interview data, in which participants reported increased confidence in performing tasks where they previously experienced freezing of gait. They also reported feeling less anxious when they experienced freezing of gait and felt better equipped with strategies to overcome freezing when it was triggered.

Given that anxiety is a risk factor and a significant predictor of developing freezing of gait [18], reducing anxiety may alleviate freezing of gait by reducing the load on attentional or cognitive resources needed to control gait in people with Parkinson disease. Our results suggest that individuals with high levels of anxiety regarding their freezing of gait may be more suited and more likely to benefit from this intervention. It also suggests that personalized rehabilitation for gait impairments is required, where individual characteristics help determine intervention modality and delivery to achieve the best outcomes [60,61].

Interestingly, 2 participants in this study (P5 and P6) specifically identified tasks and set goals to overcome their freezing in their *off* phase, as they experienced minimal freezing of gait during their *on* phase when their medication was working optimally. Even though the intervention was completed when the participants were *on*, the effects of the intervention appeared to carry over to the *off* phase. In their interviews, both participants reported that they were able to successfully implement their personalized strategies when they experienced freezing and reported achieving their goals on the Goal Attainment Scale.

Given its episodic nature and the myriad of factors that can trigger or alleviate freezing of gait, the assessment of freezing is exceptionally challenging. Common outcome measures used to assess the severity of freezing of gait, such as the Freezing of Gait Questionnaire [62] and NFOG-Q, have limitations because of their subjective nature and poor responsiveness and should be used in conjunction with other freezing of gait measures [24,31]. Several objective clinical measures of freezing of gait have been developed to complement self-report measures but freezing of gait is still not reliably captured, especially in the home environment [32]. The freezing of gait measures used in this study provided a good indication of the participants’ severity of freezing but might not be sufficiently sensitive to reflect the frequency and severity of freezing in daily life. Given that this intervention was delivered in participants’ homes with a highly personalized approach, the development of protocols and technology to measure freezing throughout the day in the home environment using wearable sensors is urgently required.

### Conclusions

Video self-modeling using an immersive virtual reality HMD plus physical practice of personalized movement strategies is a feasible and acceptable method of addressing freezing of gait in people with Parkinson disease. Future larger randomized controlled trials could explore the use of wearable sensors to measure freezing of gait at home for home-based intervention, the use of different platforms to deliver video self-modeling, and the impact of this intervention on freezing of gait, anxiety, and falls.

## Appendix

Multimedia Appendix 1 Intervention protocol.

Multimedia Appendix 2 Modified Players Experience of Need Satisfaction Questionnaire.

Multimedia Appendix 3 Semistructured interview guide.

Multimedia Appendix 4 Protocol for determining percent time frozen.

Multimedia Appendix 5 Interview themes, codes, and quotes.

Multimedia Appendix 6 Table of individual secondary outcome data.

Multimedia Appendix 7 Example of self-modeling video.

## Abbreviations

CFOG-Q: Characterizing Freezing of Gait Questionnaire
HMD: head-mounted display
NFOG-Q: New Freezing of Gait Questionnaire
PAS: Parkinson Anxiety Scale
PENS: Players Experience of Need Satisfaction
