# Supplementary material for: A Video Self-Modeling Intervention Using Virtual Reality Plus Physical Practice for Freezing of Gait in Parkinson Disease: Feasibility and Acceptability Study
Source: JMIR Form Res. 2021 Nov 3;5(11):e28315. doi: 10.2196/28315 (PMC8600439; doi:10.2196/28315)
Supplement: Multimedia Appendix 1 [file formative_v5i11e28315_app1.docx]

**Multimedia Appendix 1 - Intervention protocol**

Participants will receive six to eight home visits over six weeks, with each visit lasting approximately 60 mins.

Prior to the first home visit

- Review results of the participant’s baseline assessments. This information will assist with the assessment of the participant’s FOG triggers, identification of situations/tasks at home where FOG is troublesome and guide the development of the movement strategies.
- This includes:
- MDS-UPDRS Motor score – determine if any asymmetry between left and right is present and if the participant reports a more affected side.
- CFOGQ – identify any potential triggers and the success, or not, of any strategies used previously to reduce or overcome FOG.
- Falls history – identify circumstances of any falls and determine the risk of falls (i.e. high, moderate or low using the matrix outlined in Paul et al 2013.)
- 10m walk test – determine cadence

At the first home visit

- Participants will identify a situation in their home where FOG is troublesome.
- Use the participant’s responses on the CFOGQ to guide assessment of their FOG triggers.
- Work with the participant to develop an effective movement strategy to help them overcome their FOG in the chosen situation. These strategies may include person-specific visual cues or auditory cues, or movement strategies such as shifting weight from side to side, marching on the spot or turning in a wide arc.
- Supervise the participant as they practise the strategy.
- Once the participant successfully implements their strategy, film their best performance with the 180 degree camera.
- Check that the video is of good quality (i.e. video is smooth and clear, video captures participant’s lower limbs when performing the strategy). Film the participant again if necessary.
- Explain to the participant that they should not practice until after the video has been delivered at the next session and the therapist has supervised both the video viewing and physical practice.
- Document the following: the task completed, the environment in which the task is completed and the process in selecting the movement strategy, included the reasons why other strategies were rejected

Prior to the second home visit

- Edit the video to show the participant’s best performance of their movement strategy. Duplicate the video clip so that their performance is repeated three times in each video.
- Upload the video into the virtual reality headset.
- Watch the video using the virtual reality headset to ensure the system is operational.

At the second home visit

- Instruct the participant on how to use the virtual reality headset.
- Ensure that the participant is able to access their video using the virtual reality headset independently. If they are unable to do it independently, instruct their carer how to assist.
- Instruct the participant to view their video in sitting, twice a day, five days of the week.
- Observe the participant performing their physical practice of the strategy to ensure they are practising safely and effectively. If they are unable to perform the physical practice independently, instruct their carer how to supervise the practice.
- Instruct the participant to perform physical practice of their strategy once a day after one of the viewings, five days of the week.
- Provide the participant with their logbook. Highlight the following:
  - Virtual reality system user guide
  - Safety information for video viewing and physical practice
  - Explanation on how to complete the logbook – participants to tick the boxes to indicate when video viewings and physical practice are completed
  - Record any effects of intervention
  - Record any adverse events
  - Contact details if participants have any questions or experiences an adverse event
  - Date, time and location of assessment sessions and home visits
- Document outcomes of the home visit.

At subsequent home visits

- Review the participant’s progress with the video viewings and physical practice of the first movement strategy.
- The participant will identify a second situation where FOG is troublesome. This may be the same task with increased complexity, the same task in a different situation or a different task altogether.
- Work with the participant to develop an effective movement strategy to help them overcome their FOG in the second situation. This may be the same movement strategy used in the first situation or adjusted depending on circumstances.
- Supervise the participant as they practise the second strategy.
- Once the participant successfully implements their second strategy, film their best performance with the 180 degree camera.
- Edit the second video of the participant to show their best performance of their movement strategy. Duplicate the video clip so that their performance is repeated three times.
- Upload the second video into the virtual reality headset and ensure the system is operational.
- Ensure the participant is able to access the first and second videos using the virtual reality headset independently or with the assistance of their carer.
- Instruct the participant to view their second video in sitting, twice a day, four days of the week. In addition, instruct the participants to view their first video in sitting, twice a day, one day of the week.
- Observe the participant performing their physical practice of the second strategy to ensure they are practising safely and effectively. If they are unable to perform the physical practice independently, instruct their carer how to supervise the practice.
- Instruct the participant to perform physical practice of their second movement strategy once a day after viewing the second video, four days of the week. In addition, instruct the participant to perform physical practice of their first movement strategy once a day after viewing the first video, one day of the week.
- Review the participant’s viewing and physical practice records. Remind the participant to complete the logbook.
- Document outcome of the home visit.
- If the participant is progressing well, repeat this process with a third situation/movement strategy until the end of the six weeks intervention period.

**Examples of situations where freezing of gait is likely to occur and potential strategies to overcome freezing of gait**

| **Situations where freezing of gait is likely to occur**  People with freezing of gait may experience freezing in one or more of these situations. |
| --- |
| Initiation of gait  Walking  Turning  Stopping  Changing between non-continuous tasks (e.g. walking, stopping, and starting to walk again)  Physical environment (e.g. clutter, doorways)  High levels of anxiety |
| **Potential strategies to overcome freezing of gait**  The potential strategies to address freezing of gait outlined below was based on previous work by Nieuwboer et al in 2008, Pelosin et al in 2010, Ehgoetz-Martens et al in 2018, Ginis et al in 2018. These strategies may be used on their own or in combination with other strategies. |
| Cueing strategies   - Counting (e.g. 1-2-1-2 or left-right-left-right) - Shifting weight or stepping from side to side at a regular rhythm - Stepping to the beat of a metronome (typically set at lower than usual cadence) or music with an appropriate beat - Stepping in time with imagined metronome or musical beat - Visual cues (e.g. lines appropriately spaced on the ground for walking or turning in a wide arch, or a spot on the floor for initiating walking) - Cue cards (e.g. “long steps, lift knees”)   Movement strategies   - Shifting weight from side to side - Stepping on the spot - Stepping backwards or sideways before stepping forwards - Stepping to turn on the spot, instead of pivoting - Walking sideways instead of walking forwards - Pretending to climb up stairs (e.g. high knees)   General strategies   - De-clutter - Minimise distractions - Avoiding dual-tasking/Simplifying tasks - Take a few deep breaths before initiating task - Focusing on something else other than your feet (e.g. cue or movement)   Any other strategy suggested by the person with freezing of gait that worked for them e.g. pointing in the direction of the first step. |
| **Potential progressions** |
| Build up complex physical and anxiety-inducing environments more likely to trigger FOG (e.g. waiting for and entering a lift which has time constraints)  Build up task complexity (e.g. dual tasking such as carrying a laundry basket while walking through a door which requires opening) |

*References:*

*Paul S et al. Three simple clinical tests to accurately predict falls in people with Parkinson’s disease. Mov Disord 2013. 25:655-662.*

*Nieuwboer et al. Cueing Gait and Gait-related Mobility in Patients with Parkinson’s Disease. Topics in Geriatric Rehabilitation 2008. 24(2):151-165.*

*Pelosin et al. Action Observation Improves Freezing of Gait in Patients with Parkinson’s Disease. Neurorehabil Neural Repair 2010. 24(8):746-752.*

*Ehgoetz Martens et al. Evidence for subtypes of freezing of gait in Parkinson’s disease. Mov Disord 2018. 33:1174-1178.*

*Ginis et al. Cueing for people with Parkinson’s disease with freezing of gait: A narrative review of the state-of-the-art and novel perspectives. Ann Phys Rehabil Med 2018. 61(6):407-413.*
