# Supplementary material for: A Video Self-Modeling Intervention Using Virtual Reality Plus Physical Practice for Freezing of Gait in Parkinson Disease: Feasibility and Acceptability Study
Source: JMIR Form Res. 2021 Nov 3;5(11):e28315. doi: 10.2196/28315 (PMC8600439; doi:10.2196/28315)
Supplement: Multimedia Appendix 2 [file formative_v5i11e28315_app2.docx]

**Multimedia Appendix 2 - Modified Players Experience of Need Satisfaction (PENS) Questionnaire**

The modified PENS questionnaire used in this study was adapted from the questionnaire developed by Ryan et al in 2006. It consisted of 18 questions across 6 categories that assessed the participants’ experience of using the virtual reality system. These categories were: interest/enjoyment, competence, presence/immersion, intuitive controls, future participation, and motion sickness. It was scored on a 7-point Likert scale, where 1 = strongly disagree and 7 = strongly agree (higher score better). 4 of the 19 questions (highlighted with *) were phrased in such a way that its effect direction was opposite to the others. Results from these 4 questions were reversed during analysis to allow for calculation of mean and SD.

*Instruction:*

Please reflect on your experience of using the virtual reality system (including the headset, handheld controller, and viewing of your videos) and rate your agreement with the following statements.

1 = strongly disagree 7 = strongly agree

| *Interest/Enjoyment* | 1 | 2 | 3 | 4 | 5 | 6 | 7 |
| --- | --- | --- | --- | --- | --- | --- | --- |
| I enjoyed doing this activity very much. |  |  |  |  |  |  |  |
| This activity was fun to do. |  |  |  |  |  |  |  |
| I thought this was a boring activity.* |  |  |  |  |  |  |  |
| This activity did not hold my attention at all.* |  |  |  |  |  |  |  |
| I would describe this activity as very interesting. |  |  |  |  |  |  |  |
| While I was doing this activity, I was thinking about how much I enjoyed it. |  |  |  |  |  |  |  |
| *Competence* | 1 | 2 | 3 | 4 | 5 | 6 | 7 |
| I felt competent at using the virtual reality system to watch my videos. |  |  |  |  |  |  |  |
| I felt very capable and effective when using the virtual reality system. |  |  |  |  |  |  |  |
| *Presence/Immersion* | 1 | 2 | 3 | 4 | 5 | 6 | 7 |
| When watching my videos, I felt transported to another time and place. |  |  |  |  |  |  |  |
| When watching my videos, I felt as if I was actually there. |  |  |  |  |  |  |  |
| I was not impacted emotionally by watching my videos. |  |  |  |  |  |  |  |
| Watching my videos was engaging. |  |  |  |  |  |  |  |
| I experienced feelings as deeply in my videos as I have in real life. |  |  |  |  |  |  |  |
| I experienced genuine pride when I watched my videos. |  |  |  |  |  |  |  |
| I experienced distress when I watched my videos.* |  |  |  |  |  |  |  |
| *Intuitive Controls* | 1 | 2 | 3 | 4 | 5 | 6 | 7 |
| Learning to use the virtual reality system was easy. |  |  |  |  |  |  |  |
| The virtual reality system controls were intuitive. |  |  |  |  |  |  |  |
| *Future Participation* | 1 | 2 | 3 | 4 | 5 | 6 | 7 |
| If given the opportunity, I would use virtual reality systems for the management of my freezing of gait in the future. |  |  |  |  |  |  |  |
| *Motion Sickness* | 1 | 2 | 3 | 4 | 5 | 6 | 7 |
| I experienced motion sickness while using the virtual reality system.* |  |  |  |  |  |  |  |

*Reference: Ryan, R.M., C.S. Rigby, and A. Przybylski, The Motivational Pull of Video Games: A Self-Determination Theory Approach. Motivation and Emotion, 2006. 30(4): p. 344-360.*
