# Supplementary material for: A Video Self-Modeling Intervention Using Virtual Reality Plus Physical Practice for Freezing of Gait in Parkinson Disease: Feasibility and Acceptability Study
Source: JMIR Form Res. 2021 Nov 3;5(11):e28315. doi: 10.2196/28315 (PMC8600439; doi:10.2196/28315)
Supplement: Multimedia Appendix 3 [file formative_v5i11e28315_app3.docx]

**Multimedia Appendix 3 - Semi-structured interview guide**

Participants were asked to describe their experiences of the intervention which included both viewing of their personal videos and physical practice. Interviews were conducted in person, with the exception of two participants who completed the interviews remotely due to COVID-19 (one over the phone and one over videoconference). Carers of two participants (P1 and P7) were present during the interviews and contributed to the discussion at times. Each interview lasted approximately 45 to 60 mins, were audio recorded, de-identified and transcribed verbatim by an external transcription service.

| *Attitude towards the intervention* |
| --- |
| What did you think of the intervention to assist with your freezing of gait?  What did you like/dislike about it? Why?  What did you think of using the videos viewed in the virtual reality system as a way of learning movement strategies for your freezing of gait?  What did you think of the physical practice component of the intervention?  Did your experience of watching the video have an effect on the physical practice component of the intervention? If yes, how so?  What, if anything, did you get out of the program? |
| *Ease of undertaking the intervention* |
| What are the technological devices you currently use?  Have you used virtual reality technology before? If yes, how familiar would you consider yourself with the technology?  What was your experience of using the virtual reality technology?  How easy or difficult is it to use the virtual reality system?  Did it take some time to get used to?  What advice would you give to others about using the virtual reality headset?  Do you have any suggestions to improve the videos or the experience of watching them?  How easy or difficult is it to perform the physical practice component of the intervention?  What parts of the intervention were easy/hard to do? Why?  Do you have any suggestions for how the intervention could be changed? |
| *Perceived effectiveness of and satisfaction with the intervention* |
| Were you happy with the task we chose to video and practise?  Were there other tasks that you wanted to video and practise?  When watching your videos, what aspects of your performance stood out the most?  When you were watching your video, were you able to maintain your attention on the video?  Did you feel immersed in the virtual reality environment?  Was there anything you would change to improve the immersive-ness of your video and virtual reality experience?  Do you think your experience will be same or different if you watched your video on a computer screen?  How did viewing the videos impact on your physical practice? Explain the reason why they helped or didn’t help.  Do you think the intervention help reduce your freezing of gait? If no – why? If yes – why? Did the intervention reduce your FOG when you were on, off or both? Give an example.  Would you recommend this intervention (or specific parts of the intervention) to other people with Parkinson’s disease? Why/Why not? |
| *Sustainability of the intervention* |
| Would you continue to do any part of the intervention if the equipment and professional support as delivered in this study was available? Why/Why not? |
| Any additional comments |
