# Supplementary material for: A Video Self-Modeling Intervention Using Virtual Reality Plus Physical Practice for Freezing of Gait in Parkinson Disease: Feasibility and Acceptability Study
Source: JMIR Form Res. 2021 Nov 3;5(11):e28315. doi: 10.2196/28315 (PMC8600439; doi:10.2196/28315)
Supplement: Multimedia Appendix 4 [file formative_v5i11e28315_app4.docx]

**Multimedia Appendix 4 – Protocol for determining percent time frozen for the Ziegler and turn-in-place tests**

Our protocol used to determine percent time frozen was developed in consultation with our colleagues from KU Leuven and was based on previous work by Gilat 2019 using open source software ELAN © Max Planck Institute for Psycholinguistics, Nijmegen, Netherlands.

| **Ziegler test** |
| --- |
| **Definition of a freezing of gait (FOG) episode**  FOG is an episodic symptom defined by a marked reduction or complete absence of forward progression of the feet despite the intention to walk (Nutt et al 2011).  Any Trembling, Festination, and Akinesia are tagged as FOG.  **Definition of an ineffective step**  An ineffective step is defined in relation to the patient’s effective steps during their non-FOG walking/turning within the context of each test. Any individual or combination of the following factors are taken into consideration: difference in speed, step length, foot clearance, effort or body language; or inability to lift foot completely (e.g. heel comes off but not toes, or toes come off but not heel)  Play the video of the test at normal speed before doing any tagging to determine what an effective step may be for the person in the context of the straight and turning components of the specific Ziegler test.  **What is not considered a FOG episode**  Any clear stoppage of movement in the intended direction, which cannot be labelled with certainty as a FOG episode (e.g. during transition from one subtask to another, or one direction to another, or an adjustment for balance), is considered a movement interruption and is not tagged as FOG. |

| Ziegler test time frozen (%) = | Total time frozen during Ziegler test (s) | x 100 |
| --- | --- | --- |
|  | Total duration to perform Ziegler test (s) |  |

| **Ziegler total time frozen** | | |  |
| --- | --- | --- | --- |
| **Type** | **Start** | **End** |  |
| **Trembling**  Lack of effective motion in the intended direction with trembling of the legs despite the intention to walk/turn. (Note: trembling is typically reported at 3-8 Hz. For tagging, trembling at any frequency is included.) | The moment when the foot of the participant is suddenly no longer producing an effective step in the intended direction and is displaying FOG-related features (trembling, beginning of knee movement or beginning of heel lift, without an effective step), despite the participant’s intention to continue walking/turning. | The moment of initial toe-off of the leg that showed FOG-related features when the participant is again able to perform at least two effective alternating steps (showing no FOG-related features). |  |
| **Festination**  Lack of effective motion in the intended direction with progressive shortening of step length and increase in cadence.  (Note: festination is NOT tagged during the left and right turns of the Ziegler test.) | The moment of initial toe-off when the participant is suddenly no longer producing an effective step in the intended direction and is displaying FOG-related features (progressive shortening of the steps accompanied by an increase in cadence), despite the participant’s intention to continue walking. | The moment of initial toe-off of the leg that showed FOG-related features when the participant is again able to perform at least two effective alternating steps (showing no FOG-related features). |  |
| **Akinesia**  Lack of effective motion in the intended direction without trembling of the legs despite the intention to move. There is a lack of observable movement of the leg/foot in the intended direction (clear sticking of the foot/feet while there may be movement of the trunk in the intended direction). | The moment when the foot of the participant is suddenly no longer producing an effective step in the intended direction and is displaying FOG-related features (akinesia with a lack of observable motion of the leg /foot), despite the participant’s intention to continue walking/turning. | The moment of initial toe-off of the leg that showed FOG-related features when the participant is again able to perform at least two effective alternating steps (showing no FOG-related features). |  |
| **Ziegler total test duration** | | | |
|  | **Start** | **End** | |
|  | In standing, from the moment the first intention to move forward is observed (e.g. knee starting to bend or heel starting to lift or weight shift to the stance leg.)  Note: Moving of head to look down is not considered first intention to move. For conditions with the additional tasks, the start of the trial is after the participant has the tray in both hands. | The moment that any part of the second foot strikes the ground after both feet have cleared the doorway. | |
| **Ziegler movement interruption** | | | |
|  | **Start** | **End** | |
|  | The moment when the foot of the participant is suddenly no longer moving in the intended direction with no clear trembling, festination, or akinesia. | The moment the foot lifts off the ground to resume movement in the intended direction (or the moment a FOG episode is clearly initiated) | |

Other points to note for the Ziegler test

- At the start of the test, any steps forward while still standing up are not considered as the start step.
- At transitions (e.g. transitioning from one subtask to another or changing direction), changes in speed and step length or with weight shifting from side to side are not considered FOG unless they are associated with trembling or akinesia.
- Clear stepping on the spot where the foot is clearing the ground is considered an effective step.
- If there are multiple FOG features within a single FOG episode (e.g. starts with trembling then becomes akinetic, the most prominent FOG feature is tagged)
- If the person clearly stops moving in the intended direction with no FOG features (e.g. thinks it is the end of the test or chooses to stop, but when prompted, starts again), the time between stopping and starting cannot be considered FOG as the person has no intention to move. This is tagged as movement interruption with explanation of possible cause. Any FOG on recommencing movement in the intended direction is included and should be tagged.
- If the person chooses to stop before the test is completed, this is tagged as movement interruption.
- If the person or key features (e.g. feet) are not visible in the video due to a researcher or equipment/furniture in the way, this is tagged as a visual obstruction. FOG may or may not be tagged in the visual obstruction period, depending on whether there is sufficient information available in the view to make a decision.

| **Turn-in-place test** |
| --- |
| **Definition of a FOG episode**  FOG is an episodic symptom defined by a marked reduction or complete absence of forward progression of the feet despite the intention to walk (Nutt et al 2011).  Any Trembling and Akinesia are tagged as FOG. Festination is not tagged as FOG in this test.  **Definition of an ineffective step**  An ineffective step is defined in relation to the patient’s effective steps during their non-FOG turning within the context of the 1 min trial. Any individual or combination of the following factors are taken into consideration: difference in speed, step length, foot clearance, effort or body language; or inability to lift foot completely (e.g. heel comes off but not toes, or toes come off but not heel)  Play the video of the test at normal speed before doing any tagging to determine what an effective step may be for the person in the context of the specific turn-in-place test.  **What is not considered a FOG episode**  Any clear stoppage of movement in the intended direction, which cannot be labelled with certainty as a FOG episode (e.g. during transition from one subtask to another, or one direction to another, or an adjustment for balance), is considered a movement interruption and is not tagged as FOG. |

| Turn-in-place test time frozen (%) = | Total time frozen during Ziegler test (s) | x 100 |
| --- | --- | --- |
|  | 60 |  |

| **Turn-in-place total time frozen** | | |  |
| --- | --- | --- | --- |
| **Type** | **Start** | **End** |  |
| **Trembling**  Lack of effective motion in the intended direction with trembling of the legs despite the intention to turn.  (Note: trembling is typically reported at 3-8 Hz. For tagging, trembling at any frequency is included.) | The moment when the foot of the participant is suddenly no longer producing an effective step in the intended direction and is displaying FOG-related features (trembling, beginning of knee movement or beginning of heel lift, without an effective step), despite the participant’s intention to continue turning. | The moment of initial toe-off of the leg that showed FOG-related features when the participant is again able to perform at least two effective alternating steps (showing no FOG-related features). |  |
| **Akinesia**  Lack of effective motion in the intended direction without trembling of the legs despite the intention to turn. There is a lack of observable movement of the leg/foot in the intended direction (clear sticking of the foot/feet while there may be movement of the trunk in the intended direction). In the context of the turn-in-place test, any FOG during the trial which is not trembling is classified as Akinesia. Note: Akinesia is relatively uncommon. | The moment when the foot of the participant is suddenly no longer producing an effective step in the intended direction and is displaying FOG-related features (akinesia with a lack of observable motion of the leg /foot), despite the participant’s intention to continue turning. | The moment of initial toe-off of the leg that showed FOG-related features when the participant is again able to perform at least two effective alternating steps (showing no FOG-related features). |  |
| **Turn-in-place total test duration** | | | |
|  | **Start** | **End** | |
|  | The moment the first intention to move is observed (e.g. head/shoulders/trunk starting to rotate or knee starting to bend or heel starting to lift or weight shifting to the stance leg.  Note moving of head to look down instead of rotating/turning is not considered first intention to move. | 60 secs from the moment of the first intention to move is observed. | |
| **Turn-in-place movement interruption** | | | |
|  | **Start** | **End** | |
|  | The moment when the foot of the participant is suddenly no longer moving in the intended direction with no clear trembling, festination, or akinesia. | The moment the foot lifts off the ground to resume movement in the intended direction (or the moment a FOG episode is clearly initiated) | |

Other points to note for the turn-in-place test

- At transition (changing direction from one turning direction to the other), changes in speed and step length or with weight shifting from side to side are not considered FOG unless they are associated with trembling or akinesia.
- Clear stepping on the spot where the foot is clearing the ground is considered an effective step
- If the person clearly stops turning with no FOG features (e.g. thinks it is the end of the test or chooses to stop but when prompted, starts again), the time between stopping and starting cannot be considered FOG as the person has no intention to move. This is tagged as movement interruption with explanation of possible cause. Any FOG on recommencing turning is included and should be tagged.
- If the person chooses to stop before the minute is up, this is tagged as movement interruption.

*References:*

*Gilat M, How to Annotate Freezing of Gait from Video: A Standardised Method Using Open-Source Software. Journal of Parkinson’s Disease, 2019. 9(4): p. 821-824.*

*Nutt, J.G., et al., Freezing of gait: moving forward on a mysterious clinical phenomenon. Lancet Neurology, 2011. 10(8): p. 734-44.*
