# Supplementary material for: A Video Self-Modeling Intervention Using Virtual Reality Plus Physical Practice for Freezing of Gait in Parkinson Disease: Feasibility and Acceptability Study
Source: JMIR Form Res. 2021 Nov 3;5(11):e28315. doi: 10.2196/28315 (PMC8600439; doi:10.2196/28315)
Supplement: Multimedia Appendix 5 [file formative_v5i11e28315_app5.docx]

**Multimedia Appendix 5 - Interview data: themes, codes and supporting quotes**

| **Themes** | **Examples of codes** | **Quotes illustrating codes** |
| --- | --- | --- |
| Reflections when seeing myself | Shocked and embarrassed but used it as a driving force for improvement. | *One of the things that I changed, looking at the video, my posture and my walking, struck me as pretty poor. I have to make a conscious decision, effort, to improve. -P5*  *It made me want to improve myself, 'cause I saw myself. I'm looking at myself and what was very confronting and very embarrassing to see, what... how I started off. -P8* |
|  | Unable to move beyond the disappointment. | *Well, my first impression was how bad I looked. I thought I hid my Parkinson’s fairly well. But obviously not. Or it has got to the stage where…I've got the stage where it's quite noticeable. -P3* |
|  | Unconcerned by outward appearances. | *I enjoyed seeing myself and such. I wasn't deterred by it. -P9*  *The video was taken of me doing something the right way so I was focusing on good things not on failings that I had. -P2* |
| My experience of using the virtual reality system | Operating the HMD and controller and  navigating the platform can be frustrating | *It strikes me that the virtual reality system is not foolproof, if you make a mistake in one menu or something, you have to find your way back. And any frustration like that, you don't want to carry on. -P10*  *I thought he did get quite frustrated that he wasn't able to manipulate and get the things out. And that it didn't come up the same each time that he tried to start it. But that improved over the time that he was able to do it more on his own. -P1 carer*  *Yeah, initially I wasn't getting the hand of it. I couldn't get the picture come on so there were all these buttons to press and I kept pressing the wrong buttons. And then I had to get my husband to show me how to do it. Then after, after that a few times, then I got the hang of it. -P6*  *Because I had these problems, I sort of I don’t know how to fix it so I’ll wait till (the physiotherapist) comes or ring (the physiotherapist). -P4*  *I mean it’s pretty straightforward. Just point at something and click on it and off you go. - P2* |
|  | Self-perceived confidence/competence when using technological devices | *I suppose if you were kind of technically, technically minded, then you probably would have worked it out. I feel like my daughter might have worked it out without any problem. But in my case, with any technology I'm not very good. – P6*  *Not for people who are computer literate, it's easy for them to do it. Easy to follow the instructions. There you go. -P7*  *Yeah, first off, the virtual reality equipment was difficult to use. Because I wasn't familiar with it. And I would say people at my age and that would have difficulties with using. - P8* |
|  | Technological support is needed | *Advice at the beginning. Somebody explain things, carefully. With details and doing a couple of times with the person starting from switch on and switch off and whatever. But once that is on the ground. Then you will okay. -P5*  *A video tutorial (to help with using the system). -P10* |
| The role of the virtual reality system in supporting my learning | Novelty | *I think the goggles are fun, because they're different. -P1* |
|  | Immersive | *A 3D video is better than a 2D video so. You’re more involved, you’re more into it. -P5*  *To me it was very, very real. Very very real. And, and, you know, can I say this to you? And I never got bored at all looking at it. I just... hang on, when I'm well, I can do this, I can do this. -P7* |
|  | Minimising distractions | *You’ve got visual distractions around you. I mean when you look at the screen… well like the times I’m using the computer, I didn’t look at the screen a lot of the times when I was typing stuff. You know but you look at other things, you look past the screen. Whereas with the VR, all you can look at is the video there is nothing else around you. As long as you’ve got no noise. -P2*  *I think it would be different because you haven't got that full concentration there that with the goggles, it's just there in front of you, you can't distract yourself... I've gone in when he's had the goggles, and he hasn't even seen me come in. So there is no distraction. – P7 carer*  *There're less outside influences to bother you. Because if I'm looking at a computer screen... I mean there're other things on the desk, on the table, in the background. Whereas this headset excludes all that out of the equation. -P9* |
|  | Less is more | *I don’t think it’s any better than say someone holding a phone or a video and watching, so if there is a cost to it which obviously there is quite a considerable amount of money involved. -P3*  *It was just anti-climatic. -P3*  *It would perhaps be easier for the user with devices such as the tablet which I found comfortable. -P8* |
| Developing a deeper understanding on how to manage my freezing of gait | Impact of video self-modelling on physical practice | *You got that the video of what you’re supposed to be doing as you are doing it. You tell me what to do and I didn’t have the video, I don’t know - I might think I’m doing it the right way. And you come back in a week’s time and say that’s not what I meant at all. So I’m wrong - if I have the video it shows me what I’m meant to do. -P2*  *I had a good look at it and erm try to work out how to hide the Parkinson’s. Only that I didn't want to look like that. So I wanted to be better. -P3*  *Because I visualized it, I watched it first, and it was in my head, it made it easier when I got up to move along doing the same actions. In that way it helped when I actually had to do it. I just sort of didn't have to think. Because it was like in my head. And so I just moved, you know, the way I actually visualized it. -P6* |
|  | Video self-modelling as a learning tool | *The person you can most identify with. And that's yourself. The most appropriate person is the person who's got Parkinson's themselves and is functioning. And you can see what the program is doing for you, and with you. -P9* |
|  | The value of repetitive practice | *Well, it tends to reinforce good behaviour. You look at it and you do it you do it without thinking in the end. I’m actually doing things I did in the video when I think about it and now I don’t have to think about. It just happens as normal behaviour. -P2*  *The practice, the practical component is a typical routine, repetition to change habits. As such is a classic routine. -P5*  *I think it's probably the repetitiveness that is helping you without you being aware. The fact that you have done it so many times makes it easier. -P6*  *All Parkinson’s patients have to think of what they're doing at the time. Whereas, this, this has helped, helped so much in the sense that, you don't have to think as hard as you used to do something. -P8*    *Perhaps spread out a little more. Rather than having a daily prep having two times a week or something like that. And stretch out a little bit. I think that the intensity would have to reduce, but I think it would be a beneficial thing to have it a sort of a revision from time to time. -P1* |
|  | Improving the ability to problem solve | *I think it prepares more the attitude to when (freezing of gait) happens. For instance, you don’t cut it completely or diminish. But when it happens, you get out of the situation much quicker. -P5*  *If there's a way of prompt cueing, or visual or whatever, it has to be a bonus, it has to be a positive outcome. I've seen it. And the thing is, Parkinson's patients don't see that, they're frustrated, they're getting to a point where they have a freeze or they have… they can't turn. And they can't remember how to get themselves out of it. You know, everybody's got different strategies, but a Parkinson's person doesn't seem to be able to initiate that. -P7 carer* |
|  | Finding the right treatment option | *It's a supplement to an overall approach to the whole problem. It's not a be all, end all. It's not a magic bullet. As I say, I don't believe there's any magic bullet out there that will cure, or treat, the condition, or any condition if it comes to that. There’re usually multiple, multiple choices and multiple solutions. So it is a very viable one. And I believe I got a 100% effort out of it. -P9* |
| Impact of the intervention on my daily activities | Carry-over to other activities of daily living | *We went down last couple of weekends we’ve gone around downtown caught the ferry walking on and off without a problem. I haven’t caught a bus or train for ages because getting on getting on and off was going to be an issue but I caught the ferry and had no problems at all. -P2*  *I had a lot of difficult with crowds and I find that it's easier to go to the shopping centre now, without trying to manoeuvre around people. I'm starting to flow with-with people whilst they're walking. Whereas I'd have nothing but problems with people. Like being confronted by someone walking past you and then saying., "Sorry I bumped you into the..." I find that it... That has become a little bit easier. -P8* |
|  | Carry-over to “off” period | *All these things I do before, and therefore, they, they are difficult to do because of not taking the medication. So it helped in the sense that, you know, I could do it much faster. And it became like, you know, something I'd already seen and it was in my head that I can just carry on and do it without hesitating. -P6*  *In the freezing, yes. I experienced also, improvement. I got, the times that I got more intense, or the freezing when I wake up at night and I’m not with the medicine obviously. At that moment, almost every time is freezing but I start I stomp on the floor a little bit to start walking and so I applied the technique. -P5* |
|  | Reducing anxiety and increasing confidence | *You know, this is a tool. By using it and doing things, (P7) can improve the way he does things around the house. The amount of falls he's had, I think have been reduced. His confidence has been a lot better. -P7 carer*  *I've got more confidence. I don't panic as bad as I used to. I'm not afraid to walk around the house or outside. This is a really great help. -P8* |
