# Supplementary material for: A Video Self-Modeling Intervention Using Virtual Reality Plus Physical Practice for Freezing of Gait in Parkinson Disease: Feasibility and Acceptability Study
Source: JMIR Form Res. 2021 Nov 3;5(11):e28315. doi: 10.2196/28315 (PMC8600439; doi:10.2196/28315)
Supplement: Multimedia Appendix 6 [file formative_v5i11e28315_app6.pdf]

Table 6. Individual, mean, mean difference and 95% CI (from pre-test to post-test) for freezing of gait, anxiety and mobility measures

Outcome		Participant										n	Mean ± SD	n	Mean diff. (95% CI)
		P1	P2	P3	P4	P5	P6	P7	P8	P9	P10				
**Freezing of gait**															
Ziegler TF ON (%)	Pre	55.39	0.00	6.73	0.00	21.77	0.00	17.46	0.00	0.00	4.74	10	10.61 ± 17.60	5	-4.72
	Post	36.48	0.00	-	0.00	17.07	0.00	-	-	-	-	5	10.71 ± 16.19		(-14.89 – 5.45)
Ziegler TF OFF (%)	Pre	46.56	51.41	0.00	22.22	11.69	94.45	8.10	2.80	96.01	5.38	10	33.86 ± 36.84	5	5.44
	Post	50.90	82.75	-	26.63	9.68	83.57	-	-	-	-	5	50.71 ± 33.05		(-14.14 – 25.02)
Ziegler Duration ON (s)	Pre	192.05	19.16	27.15	20.01	53.80	23.28	57.79	24.24	17.58	58.01	10	49.31 ± 52.82	5	-20.51
	Post	101.89	16.64	-	20.64	39.39	27.21	-	-	-	-	5	41.15 ± 35.03		(-69.61 – 28.60)
Ziegler Duration OFF (s)	Pre	78.26	38.22	27.17	37.05	41.48	489.04*	35.29	27.30	373.97[#]	68.00	10	121.58 ± 166.41	5	1.24
	Post	123.48	81.98	-	50.85	40.45	393.49*	-	-	-	-	5	138.05 ± 146.40		(70.29 – 72.77)
Turn-in-place TF ON (%)	Pre	61.79	0.00	0.62	0.00	1.94	0.00	18.60	0.53	16.74	6.67	10	10.69 ± 19.29	5	-6.79
	Post	22.47	0.00	-	0.81	6.48	0.00	-	-	-	-	5	5.95 ± 9.62		(-29.49 – 15.90)
Turn-in-place TF OFF (%)	Pre	51.86	83.35	0.00	27.63	5.55	90.24	14.42	2.84	89.11	4.37	10	36.94 ± 38.16	5	0.10
	Post	44.97	87.47	-	31.80	3.72	91.17	-	-	-	-	5	51.83 ± 37.35		(-5.65 – 5.86)
NFOGQ (0-28)	Pre	22	24	22	15	11	17	23	24	21	21	10	20.00 ± 4.30	9	0.44
	Post	18	15	-	27	12	18	21	21	27	23	9	20.22 ± 5.07		(-4.22 – 5.11)
CFOGQ (0-48)	Pre	23	23	30	12	14	31	35	24	17	16	10	22.50 ± 7.76	9	-0.33
	Post	21	11	-	21	9	17	29	29	36	19	9	21.33 ± 8.78		(-8.40 – 7.73)
**Anxiety**															
PAS (0-48)	Pre	14	9	10	10	11	18	29	40	3	20	10	16.40 ± 10.97	9	-4.78
	Post	13	0	-	19	12	3	17	30	7	10	9	12.33 ± 9.03		(-11.11 – 1.56)
**Mobility**															
TUG (s)	Pre	27.00	10.44	12.88	12.29	12.88	13.94	15.34	14.50	9.4	14.02	10	14.27 ± 4.83	5	2.20
	Post	33.56	10.50	-	14.84	14.94	13.72	-	-	-	-	5	17.51 ± 9.15		(-1.17 – 5.58)
TUG (dual) (s)	Pre	72.84	11.41	14.28	16.53	22.31	17.59	30.34	17.84	12.4	14.03	10	22.96 ± 18.38	5	-3.63
	Post	57.50	10.90	-	22.47	16.40	15.25	-	-	-	-	5	24.50 ± 18.90		(-13.36 – 6.09)
Walking speed (m/s)	Pre	0.9	1.43	1.25	1.37	1.43	0.96	1.06	1.19	1.43	0.92	10	1.19 ± 0.22	5	-0.02
	Post	0.79	1.46	-	1.50	1.16	1.07	-	-	-	-	5	1.20 ± 0.29		(-0.23 – 0.19)

CFOGQ = Characterising Freezing of Gait Questionnaire, mean diff. = mean difference, NFOGQ = New Freezing of Gait Questionnaire, PAS = Parkinson Anxiety Scale, TF = time frozen, TUG (s) = Timed Up and Go test (seconds), (m/s) = (metres per second).

*This participant completed the Ziegler test under one condition only.

[#]This participant completed the Ziegler test under two conditions only.
